# Rapid Identification of Material Defects Based on Pulsed Multifrequency Eddy Current Testing and the k-Nearest Neighbor Method

**DOI:** 10.3390/ma16206650

**Published:** 2023-10-11

**Authors:** Jacek M. Grochowalski, Tomasz Chady

**Affiliations:** Faculty of Electrical Engineering, West Pomeranian University of Technology in Szczecin, 70-313 Szczecin, Poland

**Keywords:** multifrequency excitation and spectrogram eddy current testing, nondestructive testing, k-Nearest Neighbors (k-NN) algorithm, eddy currents, finite element analysis, numerical simulations

## Abstract

The article discusses the utilization of Pulsed Multifrequency Excitation and Spectrogram Eddy Current Testing (PMFES-ECT) in conjunction with the supervised learning method for the purpose of estimating defect parameters in conductive materials. To obtain estimates for these parameters, a three-dimensional finite element method model was developed for the sensor and specimen containing defects. The outcomes obtained from the simulation were employed as training data for the k-Nearest Neighbors (k-NN) algorithm. Subsequently, the k-NN algorithm was employed to determine the defect parameters by leveraging the available measurement outcomes. The evaluation of classification accuracy for different combinations of predictors derived from measured data is also presented in this study.

## 1. Introduction

Both nondestructive testing (NDT) and minor-destructive testing (MDT) are diagnostic techniques used to evaluate the properties of materials or structures without causing substantial harm. NDT techniques do not cause any damage to the material or structure, whereas MDT techniques cause only minor, easily repairable damage. Visual inspection [1], ultrasonic testing [2], magnetic particle inspection [3], radiographic testing [4], thermography testing [5], and eddy current testing are examples of common NDT techniques. MDT procedures include, among others, core drilling and flat-jack testing [6]. Several industries, including construction, manufacturing, aerospace, and medicine, employ NDT and MDT techniques. They are utilized to inspect materials for flaws, evaluate the integrity of structures, and track the condition of materials over time.

### 1.1. Pulsed Multifrequency Excitation and Spectrogram Eddy Current Testing

Eddy current (EC) nondestructive testing (NDT) is a widely used technique for evaluating the integrity and properties of conductive materials. It is a non-invasive method that allows for inspecting components without causing any damage. It also allows for the inspection of elements concealed beneath a protective coating. Eddy current testing utilizes the principles of electromagnetic induction to detect flaws, measure conductivity, and assess material characteristics.

Eddy current testing (ETC) has been employed as a nondestructive testing technique in various industries, including aerospace [7], petrochemical [8], and shipbuilding [9]. The application of this technology encompasses surface inspection [10], quality inspection [11], and thickness measurements [12], among other functions. The primary benefits of this method include its capacity for accurate detection, rapid measurement capabilities, and cost-effectiveness.

The basic version of the ETC involves the utilization of coils as the excitation component through a single-frequency approach, primarily due to its straightforward implementation [13]. The efficacy of the single-frequency eddy current technique, when employed in conjunction with phase analysis, is restricted in its ability to detect surface defects in inhomogeneous ferromagnetic materials. This is because signals resulting from cracks are distorted by noise generated by the probe’s lift-off or local variations in the specimen’s permeability [14]. The results of eddy current tests indicate that the phase angles between impedance planes exhibit frequency-dependent variations, as evidenced by the impedance plane plots.

An innovative multifrequency approach, Multifrequency Excitation and Spectrogram Eddy Current Testing (MFES-ECT) was introduced in [15,16]. The system employed a multitude of testing frequencies (usually above 20) and spectrograms. It has presented novel possibilities for precisely characterizing the defects in the materials under testing.

The Pulsed Multifrequency Excitation and Spectrogram Eddy Current Testing (PMFES-ECT) technique, an extension of Multifrequency Excitation and Spectrogram Eddy Current Testing, is introduced as a new approach to nondestructive testing [17,18]. The novel approach employs excitation through periodically repeated pulses at a predetermined interval. The pulses comprise multiple waveform periods encompassing the aggregate of sinusoids possessing a chosen frequency, amplitude, and phase. This approach maintains the benefits of multifrequency excitation while producing high-energy pulses akin to those utilized in Pulse Eddy Current Testing (PECT) [19]. This technique creates suitable conditions for detecting and identifying minor subsurface defects in conductive materials.

### 1.2. Machine Learning and Artificial Intelligence in Eddy Current Testing

Recently, significant progress has been made in machine learning, leading to notable advancements across multiple domains. It has resulted in a resurgence of interest in the research of artificial intelligence (AI) and machine learning (ML). Deep learning has garnered considerable interest due to its swift commercialization and achievements in various domains, such as computer vision, speech recognition, gaming, and machine translation. These accomplishments present novel opportunities to advance nondestructive evaluation (NDE) methodologies. The authors of [20] present a comprehensive examination of the fundamental principles underlying machine learning (ML) and its interrelation with the field of statistics. This study investigates the historical utilization and methodologies of machine learning (ML) in nondestructive evaluation (NDE) while acknowledging the prevailing challenges, such as the limited availability of reliable training data. It explores current research endeavors in machine learning for nondestructive evaluation (NDE) that seek to tackle these aforementioned challenges.

The k-Nearest Neighbor (k-NN) [21,22,23,24] technique is a widely employed algorithm in machine learning and pattern recognition domains. It is a nonparametric methodology that categorizes entities or anticipates results by evaluating their resemblance to adjacent data points within a feature space. The k-Nearest Neighbor (k-NN) algorithm is a straightforward yet efficacious approach that can be utilized for both classification and regression tasks. The k-NN method has found significant applications in material characterization due to its simplicity, flexibility, and high accuracy. It has been employed for various purposes, including defect identification, material classification, and quality assessment.

A novel approach for the automated assessment of defects in a manual eddy current (EC) inspection procedure is introduced in [25]. Manual scanning is susceptible to scanning velocity fluctuations and probe placement alterations. In order to tackle this issue, this study introduces a resilient method for normalizing EC signals by utilizing non-linear filtration techniques. The feature extraction process uses normalized Fourier and complex discrete wavelet descriptors. The classification stage utilizes various classifiers, including the k-Nearest Neighbor classifier. Evaluating the proposed system’s efficacy involves conducting tests on two types of probes: a single-frequency device equipped with an absolute probe and a dual-frequency device specifically designed to test rivets in layered structures efficiently.

The authors in [26] introduce a new application of k-Nearest Neighbor interpolation to calibrate corrosion measurements obtained from a Magnetic Flux Leakage intelligent system using readings from an ultrasonic testing scan device. By applying this interpolation method, enhanced metrics are derived, which are then utilized in the integrity assessment report of the pipeline.

According to the research conducted by the authors of [27], a technique has been proposed to effectively determine the precise crack shape and size in conductive substances. The methodology entails the utilization of a nondestructive instrument that works on the principle of eddy currents in conjunction with a postprocessing system based on machine learning. The research encompasses the phases of design and tuning, subsequently leading to a comparative performance evaluation between two machine learning methodologies: artificial neural network (ANN) and support vector machine (SVM).

The task of precisely determining conductive materials’ physical characteristics and structural attributes using eddy current (EC) measurements presents a significant challenge. The variables that substantially impact the measurements include conductivity, sample thickness, and the distance between the sample and the EC sensor (referred to as lift-off). One potential approach to address this issue involves utilizing machine learning techniques. It involves training a mathematical model using data containing known responses (i.e., the parameters of interest) and predictors (i.e., the measured EC signals). Subsequently, this model can generate forecasts of the response values for a novel collection of measurements. Research paper [28] presents a novel methodology that utilizes machine learning techniques to eliminate the necessity of computationally demanding computations and empirical data to train predictive models.

The work of [29] proposes machine learning techniques for signal inversion from NDT-EC sensors. This study aims to accurately determine the dimensions and extent of defects, thereby facilitating the geometric analysis by solving the inverse problem. The impedance of the sensor-cracked part system, which represents the crack signature, was established by creating a database using 3D finite element simulations. Experimental validation was performed to ascertain the constructed database’s precision. The machine learning algorithms underwent training using the provided database. The findings indicate that the implemented methodologies can effectively measure and assess the presence of flaws.

### 1.3. Novelty and Significance of the Research

The objective of this study was to assess the viability of using numerical simulations in 3D FEM software (COMSOL Multiphysics 6.1) in conjunction with the PMFES-ECT method to gather simulated data in order to train the k-NN machine learning algorithm for quickly estimating selected defect parameters (depth and length) in conductive materials.

Both the traditional MFES-ECT and its more recent extension, PMFES-ECT, enable the acquisition of spectrograms, the interpretation of which is left to the operator in order to ascertain the defect parameters. Artificial intelligence algorithms can considerably reduce the duration required for the analysis of measurement data and the assessment of parameters related to possible defects.

As previously indicated, the utilization of artificial algorithms in Eddy Current Testing has already demonstrated successful implementation. One methodology involved the utilization of artificial intelligence algorithms for the purpose of analyzing measurement data, intending to enhance the accuracy and comprehensiveness of defect detection and evaluation. In [30], the application of different artificial intelligence (AI) algorithms was demonstrated for the analysis of eddy current signals. These signals were measured under diverse experimental conditions and involved various types of discontinuities in AISI-type 316 stainless steel sheets and plates. In a paper [31], an unbalanced weighted k-Nearest Neighbor (k-NN) algorithm was employed, which was based on the support vector machine (SVM) approach, to enhance the identification of defects in pipelines. This approach aimed to address challenges such as noise and interference that could hinder the accuracy of defect detection. In [32], the k-Nearest Neighbors (k-NN) algorithm was employed to evaluate the extent of the defect by utilizing a specialized arrayed uniform eddy current probe.

In addition to the purely experimental approach, preliminary tests on the use of numerical models were also carried out. The alternative methodology involves utilizing the analytical model of the eddy current sensor to generate data for the purpose of training the prediction models [27,33]. This approach enables the avoidance of computationally intensive tasks and eliminates the need for experimental data in the training of prediction models. Moreover, in [28], the utilization of a 3D finite element simulation for the purpose of creating a learning database was proposed. The learning database for the multi-layer perceptron network consisted of the impedance values of the sensor-cracked part system, which served as the crack signature. 

In contrast to the aforementioned prior research, our methodology incorporates the integration of numerical modeling and artificial intelligence algorithms to assess experimentally acquired signals. In this study, we propose the combination of the Pulsed Multifrequency Eddy Current Testing technique with the k-Nearest Neighbors (k-NN) algorithm for the purpose of evaluating the dimensions, specifically the length and depth, of defects in conducting plates. The acquisition of data regarding learning databases is exclusively reliant upon the utilization of three-dimensional finite element method (3D FEM) simulations. Initially, a numerical model was formulated to analyze the phenomenon under investigation. The purpose of the model was to reconstruct the laboratory setup and also to explore scenarios that were not tested in the experiment (namely the different lengths of the defects) in order to evaluate the feasibility of applying the approach in a broader context. The model comprised a replicated sensor described in previous research [15,17], in conjunction with a simulated sample containing various defects. The modeling of the sensor’s movement over the defect was carried out as well. The consistency between the simulation results and the measurement results was additionally confirmed. The simulation data was subsequently employed solely for the purpose of training the k-NN artificial intelligence algorithm. Subsequently, the aforementioned algorithm was employed to approximate the parameters related to defects within the material under study.

The conducted experiments demonstrated that the incorporation of defect and sensor modeling using the Finite Element Method (FEM), coupled with the utilization of the PMFES-ECT and k-NN techniques, can yield significant improvements in defect detection efficiency. The utilization of a numerical model that is simple to modify offers novel opportunities. It is important to highlight that the presented method enables the identification and assessment of naturally occurring, irregular defects (such as cracks), which can be easily simulated numerically. Hence, this methodology exhibits considerable flexibility and holds major practical significance.

### 1.4. Organization of the Paper

The article is organized as follows: first, a thorough description of the sample under examination; second, a description of the Pulsed Multifrequency Excitation and Spectrogram ECT Method (PMFES-ECT); third, a thorough description and properties of the used transducer; fourth, a description of the parameters and characteristics used for defect’s depth and length estimation; fifth, a description of the used k-NN algorithm with its application; and finally, results derived from the measurements and predictions of specific defect parameters, as well as a comprehensive analysis of the results.

## 2. Materials and Methods 

**Figure 1:**
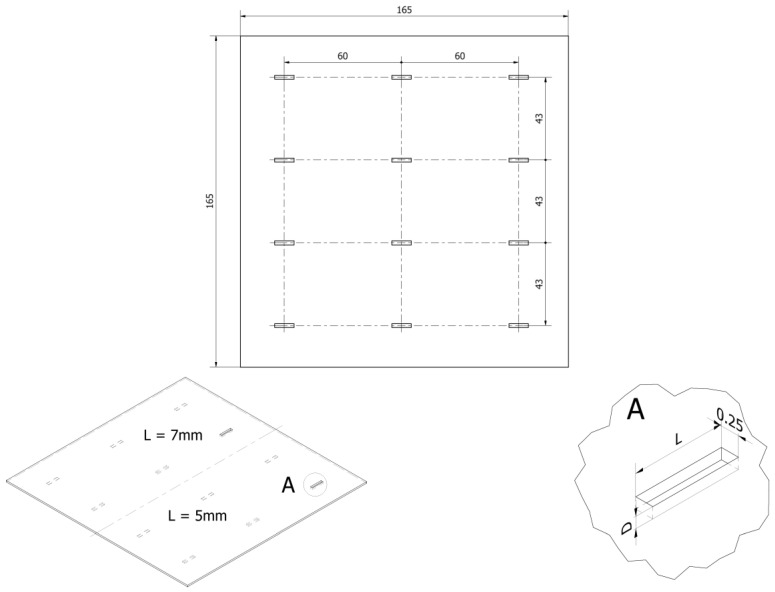
The view of the specimen with the notches of the length 5 mm and 7 mm (D denotes the defect depth, L denotes the defect length).

The object under examination was a plate made of INCONEL600 (Nippon Steel Corporation, Tokyo, Japan) that had been intentionally flawed using an electric discharge method. The plate was 1.25 mm thick, 165 mm long, and 165 mm wide. Twelve artificial defects were manufactured in the plate. The notches were 5 mm or 7 mm long, 0.25 mm wide, and depth ranged from 0.125 mm to 1.25 mm. The specimen with the defects (notches) and a close-up of the single notch with dimensions are shown in Figure 1.

Table 1 contains the properties of the utilized sample made of INCONEL 600.

The defect depth is measured from the underside of the specimen. Unless the notch was cut through, measurements were taken from the opposite side of the defect—the top side of the plate. All parameters of the defects are listed in Table 2. 

### 2.1. Pulsed Multifrequency Excitation and Spectrogram ECT Method (PMFES-ECT) and Information Extraction

The eddy current technique is used in this work to detect and characterize defects. The magnetic field generated in the excitation coils penetrates the subject material up to a specific depth depending on the frequency, configuration, and material parameters. Under the influence of this field, eddy currents are induced in the tested material, and a detectable secondary magnetic field is produced.

A novel extension of Multifrequency and Spectrogram Eddy Current Testing [9] is the Pulsed Multifrequency Excitation and Spectrogram Eddy Current Testing method. Excitation in the form of pulses is used in this method. Each pulse consists of several waveform periods. The waveform is achieved by adding sinusoids with different frequencies, amplitudes, and phases. The period length was selected experimentally to eliminate the transient state in the response signal and obtain a proper frequency resolution for subsequent analysis.

Combining numerous sinusoidal waveforms generates an exciting multifrequency signal. This signal general formula is as follows:

(1) $$u_{exct}\left(t\right)=\overset{n}{\underset{i=1}{\sum }}a_{i}U_{i}\sin\left(2\;\pi f_{i}+\phi_{i}\right)$$

where $U_{i}$ is the amplitude of the *i*-th sinusoid, $a_{i}$ is normalization factor $f_{i}$ is frequency and $\phi_{i}$ is the phase angle of *i*-th sinusoid. The factor $\phi_{i}$ is calculated using the formula:

(2) $$\phi_{i}=\pi \frac{i^{2}}{N}$$

where *N* is the total number of sinusoids. Setting the parameter $\phi_{i}$ reduces the crest factor of the signal and thus improves the power delivery to excitations coils.

The adjustment/normalization of the excitation signal is performed to ensure a constant amplitude across the entire spectrum of the signal from the pickup coil. If the amplitudes $U_{i}$ of the harmonics are the same for all frequencies, then the amplitudes of successive harmonics in the measured signal will not be constant. It results from the variable impedance of coils at different frequencies, signal attenuation, the inductance of connection cables, and parasitic capacities. To counteract this, we initially measured the response to the excitation with the same amplitudes of all harmonics $U_{i}$ and the transducer placed over a homogeneous part of the material. Then, we calculated the correction coefficients $a_{i}$ for successive $U_{i}$ values according to the formula:

(3) $$a_{i}=\frac{U_{ref}}{U_{i\;measured}}$$

where *U_ref_* is the reference value we want to acquire and *U_i_* measured is the current value of the amplitude of successive components. The *U_ref_* value is selected not to exceed the maximum sensor operating current. The normalization factor (3) is then used to generate a new final excitation signal.

Such an excitation signal driving the transducer’s excitation coils induces eddy currents in the tested sample, resulting in the appearance of a detectable secondary magnetic field. The resulting signal acquired from the pickup coil from the transducer is subjected to additional processing. First, the FFT decomposition was performed on each measured raw signal, and successive frequencies’ amplitudes were determined. The differential amplitude for each frequency and each measurement point is then computed as the difference between the actual amplitude measured at the measurement position and the amplitude measured at the uniform, defect-free material location:

(4) $$\Delta U_{f}=U_{f}-U_{0,f}$$

where $U_{f}$ is the signal amplitude for a given frequency f at a given measurement point and $U_{0,f}$ is the signal amplitude for a given frequency f at a uniform, defect-free part of the material. Afterward, the linear trend was eliminated from the data. Finally, a Butterworth low-pass filter was applied to the achieved differential signals. 

The presentation of the signal measured from a pickup coil as a spectrogram [9] is a crucial aspect of the PMFES-ECT method. The spectrogram is a three-dimensional representation of the relative amplitude of a signal’s frequency components as a function of sensor position [9]. Selected parameters of the spectrogram can be calculated and effectively used to evaluate the parameters of defects.

The following spectrogram parameters are used to estimate defect depth and length:

1. $S_{MAX}$—a maximum value of the spectrogram,
2. $X_{MAX}$—a position for which the spectrogram achieves the maximal value,
3. $f_{MAX}$—a frequency for which the spectrogram achieves the maximal value.

In addition, the following spectrogram-derived features are used:

4. $S{\left(f\right)}_{x=X_{MAX}}$—a frequency characteristic at the point $x=X_{MAX}$, where the spectrogram reaches the maximal value,
5. $S{\left(x\right)}_{f=f_{1},f_{2},\ldots ,\;f_{15}}$—an amplitude characteristic for each frequency versus sensor position,
6. $L_{f=f_{1},\;f_{2},\;\ldots ,\;f_{15}}$—derived from the characteristic $S{\left(x\right)}_{f=f_{1},f_{2},\ldots ,\;f_{15}}$, the parameter is calculated using Equation (5) for each frequency.

For each successive frequency, the parameter $L_{f=f_{i}}$ is derived from the curve $S{\left(x\right)}_{f=f_{i}}$ using the formula:

(5) $$\begin{array}{c} L_{f}=\max X_{f}-\min X_{f}\\ \mathrm{where}\;X_{f}=\left\{x\;:\frac{S{\left(x\right)}_{f}}{\max S{\left(x\right)}_{f}}>0.1\right\} \end{array}$$

**Figure 2:**
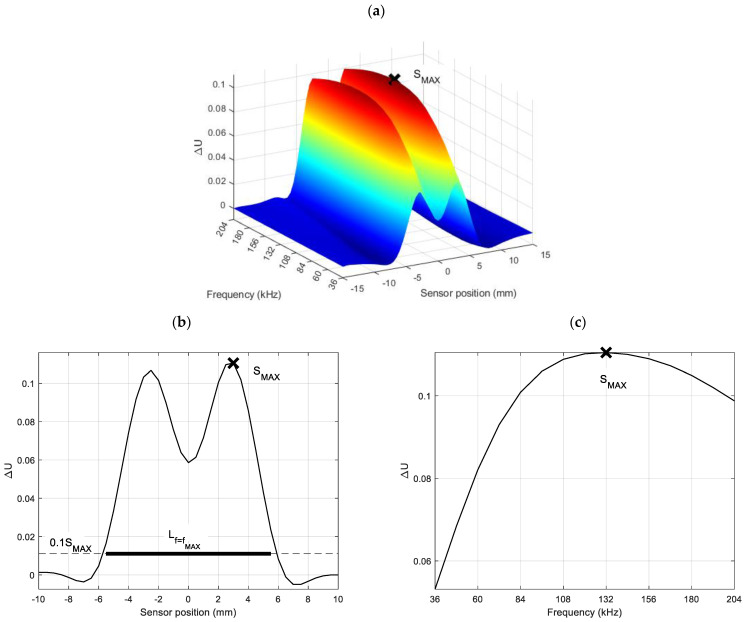
The view of the sample spectrogram derived from measurement data, (**a**) the three-dimensional representation of the spectrogram, featuring a designated point marked as $S_{MAX}$, (**b**) an amplitude characteristic $S{\left(x\right)}_{f=f_{MAX}}$ corresponding to the frequency $f_{MAX}$ with respect to the position of the sensor with the denoted $L_{f=f_{MAX}}$ value (maximum distance between points that reach at least 10% of the maximum $S_{MAX}$ value for the $f_{MAX}$ frequency), (**c**) a frequency characteristic $S{\left(f\right)}_{x=X_{MAX}}$ at the position $x=X_{MAX}$, where the spectrogram reaches the maximal value $S_{MAX}$.

An example of the spectrogram is shown in Figure 2, wherein the $S_{MAX}$ has been marked. The figure shows also the characteristics of $S{\left(f\right)}_{x=X_{MAX}}$ for the $X_{MAX}$ position and $S{\left(x\right)}_{f=f_{MAX}}$ for the frequency $f_{MAX}$ with indication of the $L_{f=f_{MAX}\;}$ parameter for this frequency. Consequently, these parameters were utilized to predict the size of the flaw in terms of its depth and length.

### 2.2. Detailed Description and Properties of the Transducer

**Figure 3:**
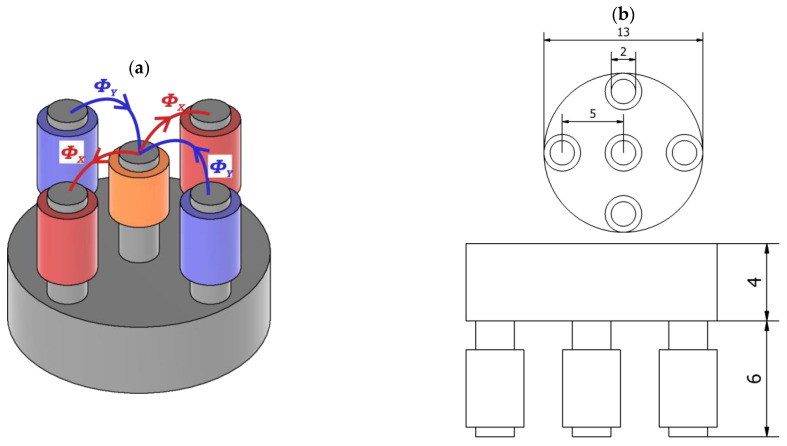
The view of the transducer. A pickup coil is positioned on the middle leg, (**a**)—a view showing magnetic fluxes from excitation coils ($\Phi_{x}$ from one set of coils, $\Phi_{y}$ from another set of coils), (**b**)—a bottom and front view with dimensions.

The simplified view of the transducer [15] is provided in Figure 3a.

The pickup coil on the center column of the five-column ferrite core detects the differential flux produced by two oppositely oriented pairs of excitation coils. Flux created in the pickup coil by one pair of excitation coils flows in the opposite direction as flux produced by the other pair. In the case of uniform material, the resulting flux in the pickup coil is close to zero. A signal occurs on the measuring coil when a flaw in the tested specimen shows up.

The relative maximum permeability of the ferrite core is $\mu_{r}=1000$. A multifrequency signal generated by the arbitrary wave generator amplified by a high-frequency power amplifier drives the exciting coils. Figure 3b show the sensor’s dimensions and Table 3 shows the transducer parameters.

**Figure 4:**
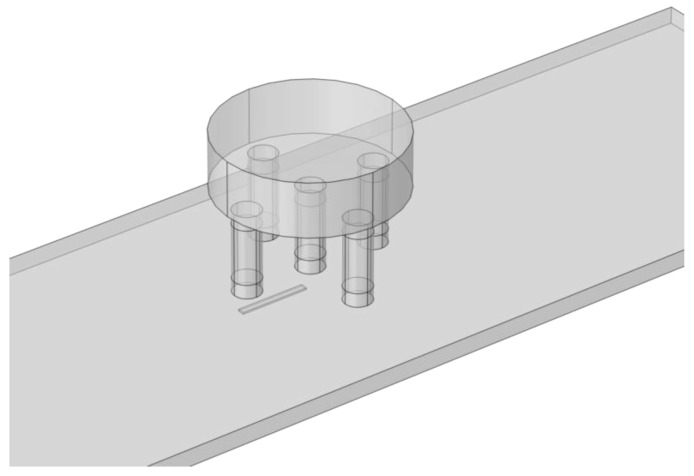
Transducer view above the tested specimen with a defect.

As shown in Figure 4, the transducer is positioned over the examined material, touching its surface with a lift-of equal to 0.3 mm. The XY linear positioning system moves the transducer along the defect in increments of 0.5 mm, ranging from −20 mm to 20 mm from the defect’s center.

### 2.3. Measurement System

**Figure 5:**
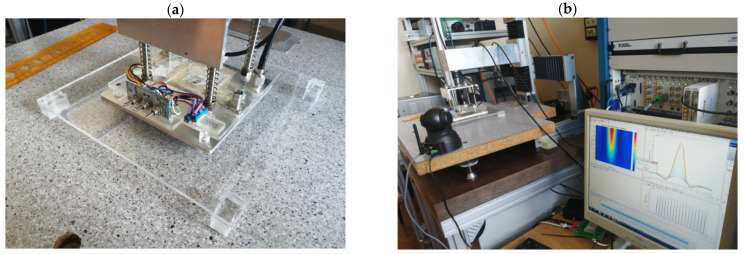
The view of the measurement system, (**a**)—a close-up of the measuring sensor, (**b**)—the entire system with a measuring computer and an XY scanner.

The measurement system (shown in Figure 5) encompasses an excitation signal generator (NI PXI 5422, manufactured by NI and based in Austin, TX, USA). This generator possesses a sample rate of 200 MS/s, an 80 MHz bandwidth, and a 16-bit DA converter. A power amplifier (HSA 4101, manufactured by NF Corporation and based in Yokohama, Japan) is also used as the excitation coils’ driver. The power amplifier operates within a frequency range of direct current (DC) to 10 MHz, with a slew rate of 5000 V/μs, a maximum current of 1.4 A, and an amplification gain ranging from 1 to 20.

The pickup coils of the transducer are connected to the analog-to-digital (A/D) capture device (NI PXI 5922, manufactured by NI in Australia and the United States) via the signal amplifier (Krohn-Hite model 3988). The highest sampling rate of the A/D converter is 15 MS/s, and its maximum resolution is 24 bits. Lastly, a personal computer (PC) equipped with dedicated software programmed in the MATLAB environment completes the setup.

The excitation signal generator produces pulsed signals transmitted to the power amplifier. The power amplifier then supplies these signals to the excitation coils within the transducer, thereby generating eddy currents within the specimen undergoing testing. The pickup coil within the transducer measures the resultant magnetic field, comprising contributions from excitation signals and the eddy currents within the material being inspected. After amplification by the signal amplifier, the A/D converter captures the voltage induced in the measuring coil and stores it in the computer for further analysis. The transducer is incrementally moved to the next measurement point using an XY linear positioning system, with steps of 0.5 mm as previously mentioned. Ultimately, the PC software (Mathworks MATLAB R2023a with custom scripts) oversees the entire system’s operation, coordinating the various components and managing data acquisition and processing.

### 2.4. Spectrogram Parameters and Characteristics Used for Defect Depth Estimation

Four parameter groups have been proposed to estimate the depth of a defect. The data is displayed in Table 4 as follows.

The D-1a group relies on parameters that are obtained through a direct reading of the spectrogram ($f_{MAX}$—a frequency for which the spectrogram achieves the maximal value, $S_{MAX}$—a maximum value of the spectrogram).

The parameters in the D-1b group are achieved from interpolation. The characteristic $S{\left(f\right)}_{x=X_{MAX}}$ was first approximated through a third-degree polynomial function, and subsequently, the parameters $f_{MAX}$ and $S_{MAX}$ were extracted from the resultant curve.

The D-2 group employs identical parameters to those of D-1b, with the exception that $\log\left(S_{MAX}\right)$ is utilized in place of $S_{MAX}$.

The depth estimation technique employed by the D-3 group involves the utilization of α and γ parameters. The method of obtaining them involves the approximation of the characteristic $S{\left(f\right)}_{x=X_{MAX}}$ through the utilization of the following approximation function [29]:

(6) $$S\left(f\right)=\alpha f^{2}e^{-\sqrt{f\gamma }}$$

A reference database for each group of predictors was calculated using simulation data obtained from FEM analysis. The databases in question contained individual records that included information about the length and depth of the defect, as well as the appropriate parameters for each respective group. Ultimately, four databases were procured for each of the groups, and each database consisted of forty-two records, encompassing seven lengths and six depths per record.

Similarly, a database containing measurement data for each group was created. Each database mentioned above comprised twelve distinct records for 5 mm and 7 mm lengths and six different depths for each length.

### 2.5. Spectrogram Parameters and Characteristics Used for Defect Length Estimation

Analogous to estimating depth, reference databases were also created to estimate the length of defects. Four distinct sets of predictors were proposed to estimate the length of defects. The groups are presented in tabular format (Table 5).

The L-1 group employs the frequency f parameter and the corresponding parameter $L_{f}$ for each frequency. Therefore, a single defect generates a number of distinct database records equal to the number of used frequencies.

Similar to the L-1 group, the L-2 group employs the $L_{f}$ and frequency f parameters along with the depth of the defect D. The determination of the depth of a defect D involves an initial estimation of said depth, which is subsequently utilized as a parameter for the estimation of its length.

As a predictor, the L-3 group employs a vector composed of *L_f_* values for all frequencies $L=\left\{L_{f_{1}},L_{f_{2}},\ldots ,L_{f_{15}}\right\}$ that correspond to a specific defect.

The L-3 group is expanded by the defect depth parameter D to form the L-4 group.

Two reference databases were subsequently acquired. Each database record for the L-1 and L-2 groups included the defect length, the defect depth D, the frequency f, and the $L_{f}$ parameter. There were 630 records altogether in this database.

The length, depth of the defect D, and $L_{f}$ parameters vector L were all included in the reference database for groups L-3 and L-4. There were 42 records altogether in this database.

Databases for the measured data were made similarly. This database had 180 records (two lengths, six depths, and fifteen frequencies) for groups L-1 and L-2. The database included 12 records for groups L-3 and L-4 (two lengths and six depths).

### 2.6. The k-Nearest Neighbours Algorithm

The defect parameters (depth, length) were predicted using the k-Nearest Neighbors algorithm. For every depth predictor group (D-1a, D-1b, D-2 and D-3) and length predictor group (L-1, L-2, L-3 and L-4) separate tables with chosen predictors were generated. The z-score (7) was used to normalize each predictor:

(7) $$z=\frac{x-\mu }{\sigma },$$

where x—is a raw predictor parameter, μ is the mean of the parameter from the whole table for the selected predictor, and σ is the standard deviation of the selected predictor.

The choice of a suitable distance metric in the k-Nearest Neighbors (k-NN) algorithm was impacted by varied predictor variables. The suitability of the scaled Euclidean distance measure was considered in this context. In addition, identifying the most suitable scale parameters for each predictor and determining the optimal number of neighbors required using Bayesian optimization methodologies.

The training and test datasets were stratified by randomly partitioning the simulation dataset. These partitions were then employed in k-fold cross-validation to ensure an unbiased assessment of the classification model. The classification loss function, selected as the misclassified rate in decimal format, served as a quantitative measure for evaluating the model’s performance:

(8) $$loss=\overset{n}{\underset{k=1}{\sum }}w_{k}I\left\{\hat{y_{k}}\neq y_{k}\right\},$$

where $\hat{y_{k}}$ is the class label corresponding to the class with the maximal score, $y_{k}$ is the observed class label, $w_{k}$ is the normalized weight for observation k and I is an indicator function.

Due to the number of used frequencies, multiple length estimates were derived for each individual defect in the case of defect length estimations in groups L-1 and L-2. The ultimate estimated defect length was determined as the most frequently occurring value among these results. The loss function was computed in a manner suitable for this particular case.

The classification model for the raw measured data was constructed using the results obtained from the parameter optimization process, focusing on the scale and number of neighbors. The classification outcomes for this dataset are presented in Section 5.

### 2.7. 3D FEM Simulation Model

**Figure 6:**
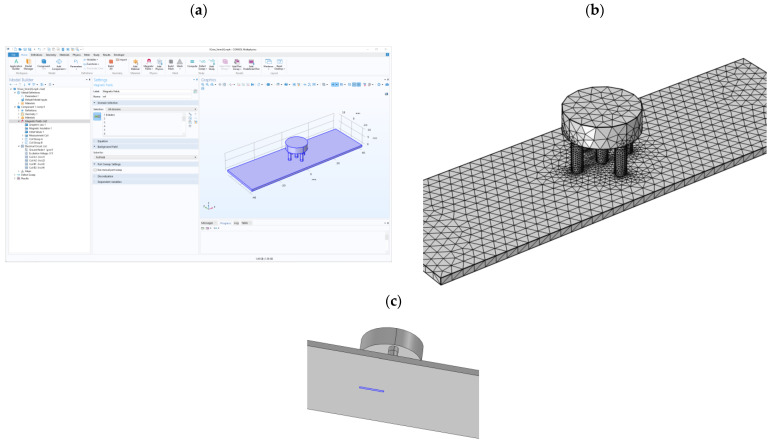
View of the simulation environment, (**a**)—view of the COMSOL Multiphysics software with a module list, (**b**)—view of the finite element mesh, (**c**)—the enlarged view of the specimen with the defect (indicated by blue color).

In order to generate reference data for the k-Nearest Neighbors (k-NN) method, a simulation was conducted utilizing a three-dimensional model (3D) of the eddy current sensor and a plate containing defects of varying lengths and depths. This simulation was performed within the COMSOL Multiphysics 6.1 software (Figure 6a), utilizing the AC/DC module (particularly the Magnetic Fields—mf and Electrical Circuits—cir submodules). In each separate simulation, a single defect (with a fixed depth and length) was simulated. About 70,000 (Figure 6b) mesh elements comprised the 3D model of the sensor with the tested specimen. The excitation and pickup coils were simulated as boundary conditions (described as Boundary->Coil in COSMOL) on sections of the sensor. The defect was simulated as a domain with air parameters (i.e., 1 S/m resistivity).

**Figure 7:**
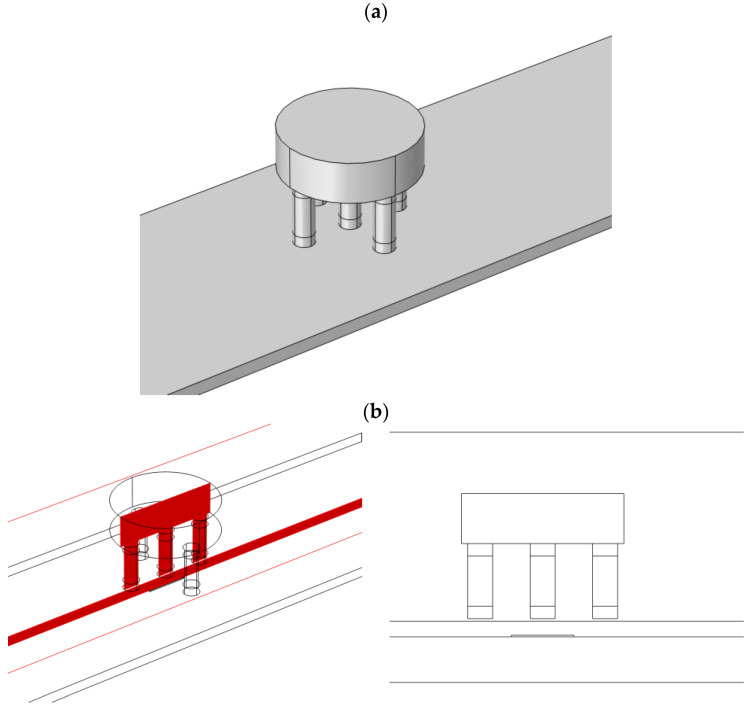
The geometry of the eddy current sensor and specimen with the defect, (**a**) a three-dimensional view of the configuration, (**b**) a cut plane through the intersection, and a two-dimensional view (the flaw is visible on the bottom of the specimen under the sensor).

The simulations were conducted in the frequency domain for specific frequencies, the same as the actual measurements. Figure 7 illustrates a close-up of the sensor model and the test sample.

The plate in the simulation had dimensions of 80 by 25 mm and a thickness of 1.25 mm (equivalent to the actual sample’s thickness). The plate conductivity is set to 1 MS/m, and the relative permeability is set to 1 (Table 6). The conductivity of the ferrite core is σ=1 S/m, while the relative magnetic permeability is $\mu_{r}=1000$. 

Defects ranging in length from 1 to 7 mm with 1 mm increments were simulated. For each of these lengths, simulations were conducted for various defect depths ranging from 10% to 100%. The information is depicted in Table 7.

For each defect length and depth, a series of simulations were conducted by moving the defect relative to the sensor from −25 mm to 0 mm (the midpoint directly below the sensor) in increments of 1 mm (in the range of −25 mm to −10 mm) and 0.5 mm (in the remaining range). 

A total of 630 simulations were performed (seven lengths, six depths, and fifteen frequencies). Based on these data, a database containing data for the k-NN algorithm was created (as explained in Section 2.4 and Section 2.5).

**Figure 8:**
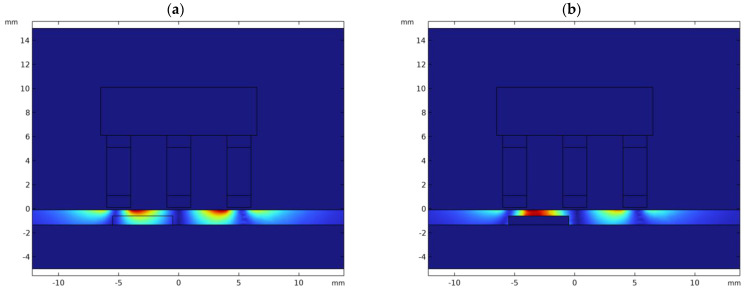
View of the distribution of eddy currents (excitation frequency 48 kHz) in the cross-section of the sample in case of (**a**) no defect, (**b**) presence of the defect (length 5 mm, depth 60%).

A distribution (cross-section view) of eddy currents (for the excitation frequency of 48 kHz) in the tested material without and with a defect (depth of 60%, length of 5 mm) is shown in Figure 8.

**Figure 9:**
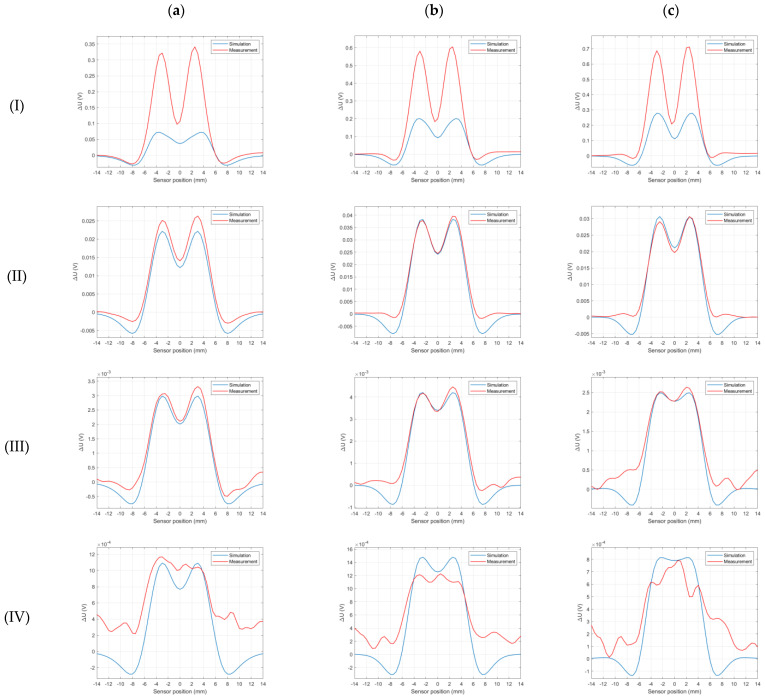
Comparison of measured and simulated signals for selected frequencies and defect depths. Blue indicates simulation signals, while red indicates measured signals. The columns show frequencies: (**a**)—48 kHz, (**b**)—120 kHz, (**c**)—192 kHz, and the rows show depths: (**I**)—100%, (**II**)—60%, (**III**)—20%, (**IV**)—10%.

Figure 9 below shows a comparison of the signals obtained from the simulation and the signals measured for selected frequencies and depths. For the majority of depths, the simulations accurately reflect the shape of the waveforms. Compared to simulations, real measurements of the deepest defects exhibit a significant amount of noise and significant waveform distortions. For a defect depth of 100% (cut-through), however, the measured signal’s amplitude is more than twice as large as that predicted by the simulation.

## 3. Experimental Results

A trained classification model was employed to predict defect parameters, precisely the depth and length, in a plate made of INCONEL that included artificially generated flaws, as outlined in Section 2. The measurement results are categorized according to the defect’s depth and length.

### 3.1. Defect’s Depth Assessment Using the k-NN Algorithm

**Figure 10:**
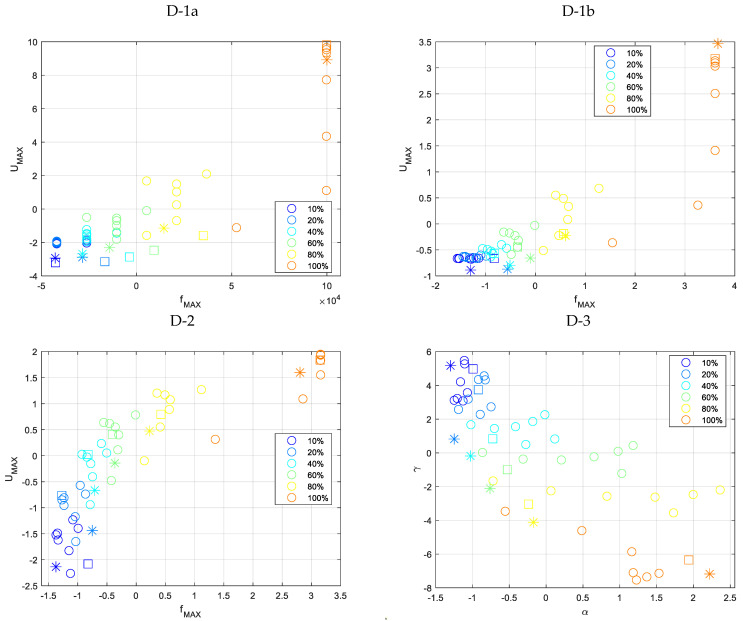
View of depth estimation cluster data plots for various groups of predictors. The color indicates the defect’s depth, while the circles (O) represent simulation (learning) data, the stars (*) represent measured data for a 5 mm defect, and the squares (□) represent measured data for a 7 mm defect. All presented data have been normalized by z-score.

Cluster data plots for different groups of predictors are depicted in Figure 10. It displays simulation data (used to train the k-NN algorithm) and measurement data plotted for two defect length types (5 and 7 mm).

Table 8 compares the loss function values for the cross-validation classification model (C-V CM) and the prediction classifier losses of defect depth in the tested specimen, categorized by different groups of predictors.

**Figure 11:**
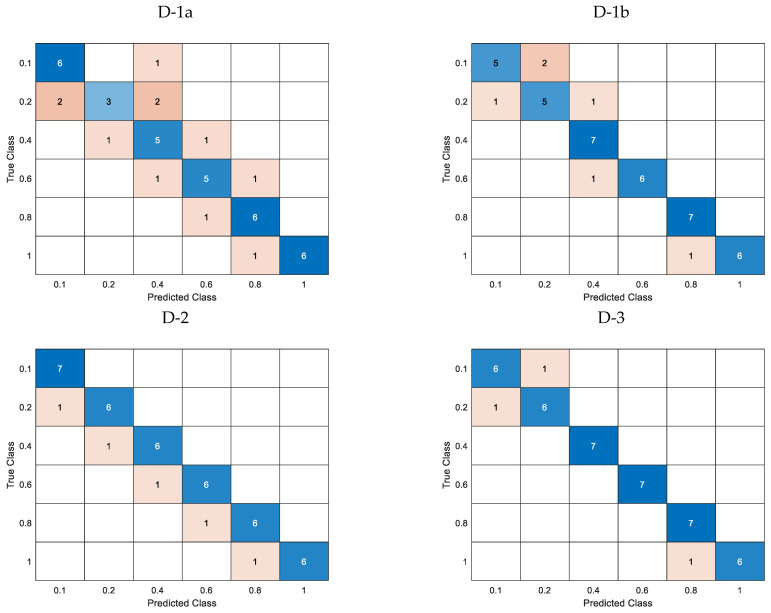
Confusion matrices for the cross-validation classification model utilizing simulation data for different depths and predictor groups.

**Figure 12:**
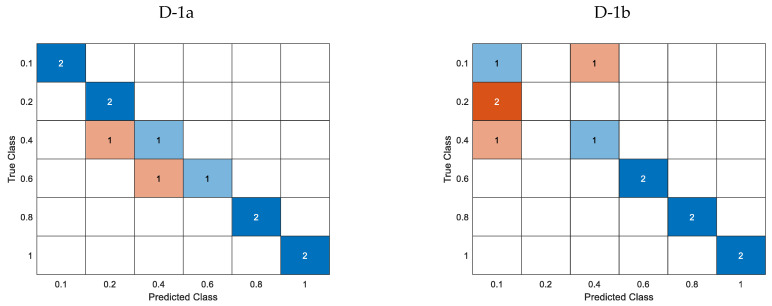

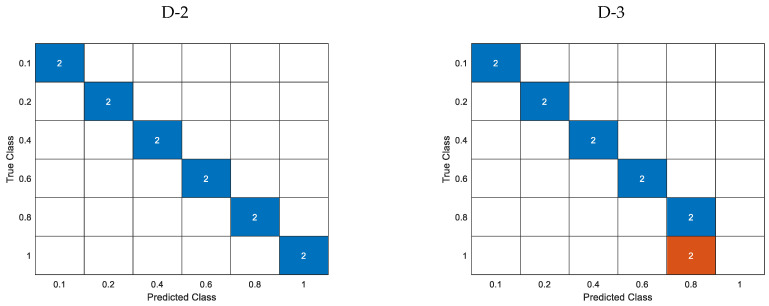
Confusion matrices for the trained k-NN classification model utilizing measured data for different depths and predictor groups.

The confusion matrices for the cross-validated classification model and the trained k-nearest neighbor classifier model are shown in Figure 11 and Figure 12, respectively.

Table 9 displays the $R^{2}$ regression coefficient for the fitting of the curve to both simulation (FEM) and measurement (MEAS) data across various depths and groups of predictors. The predictor values were calculated using the curves per the guidelines outlined in Section 2.4.

### 3.2. Defect’s Length Assessment Using the k-NN Algorithm

**Figure 13:**
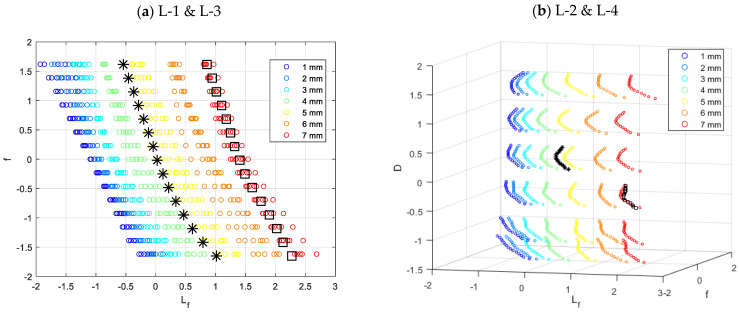
View of cluster data plots for length estimation for various groups of predictors. The color represents the defect’s depth, while the circles (O) are simulation (learning) data. The asterisks (*) represent data for a 5 mm defect, while the squares (□) represent data for a 7 mm defect. In both groups L-1 and L-3 (**a**), the defect depth of 5 (*) and 7 (□) mm was equal to 40%. In the case of groups L-2 and L-4 (**b**), the 5 mm (*) defect depth was 40%, whereas the 7 mm (□) defect depth was 60%. Using z-score, all presented data have been normalized.

Figure 13 depicts cluster data plots for various groups of length predictors. It displays simulation data (used to train the k-NN algorithm) and measurement data for two defect length types (5 and 7 mm) plotted on a graph.

Table 10 compares the loss function values for the cross-validation classification model (C-V CM) and the prediction classifier loss of defect length in the tested specimen, categorized by different predictors. The loss function values pertaining to each frequency, considered a distinct record, have been presented in brackets for the L-1 and L-2 groups. The mode of the observed frequency values was identified as the ultimate outcome of the loss function.

**Figure 14:**
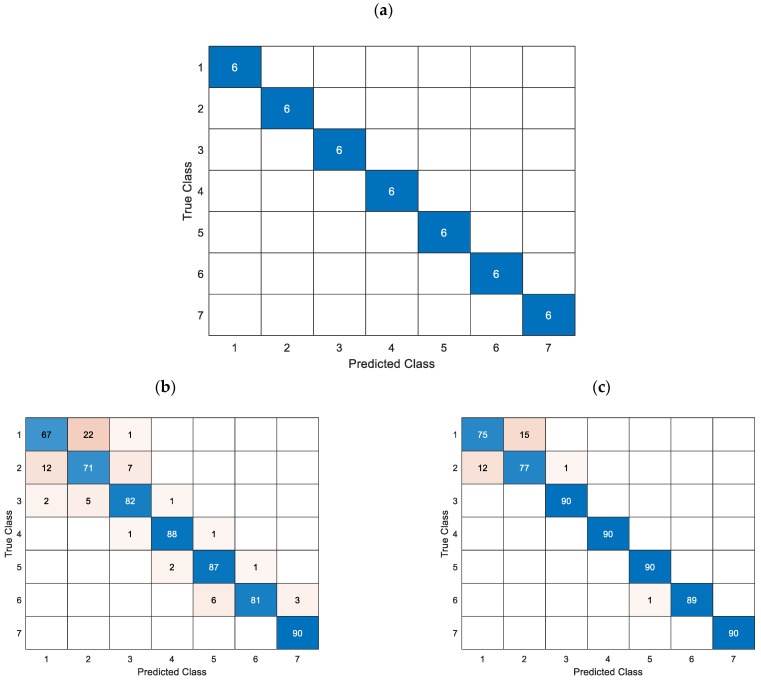
Confusion matrices for the cross-validation classification model utilizing simulation data for different lengths, (**a**)—matrix for the L-1, L-2, L-3, and L-4 predictors groups, (**b**)—matrix for the L-1 group and (**c**)—matrix for the L-2 group, treating each frequency as a distinct record.

**Figure 15:**
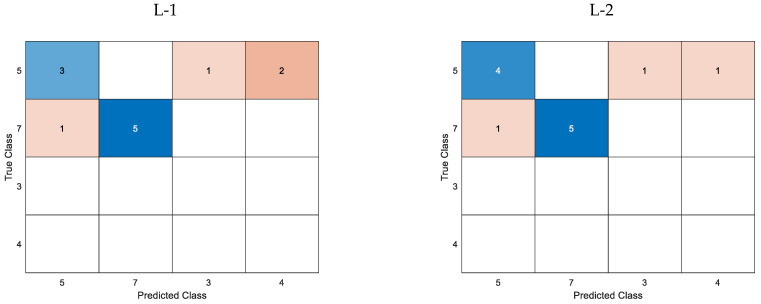

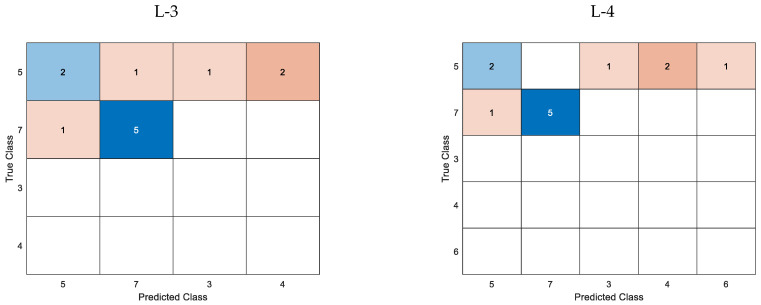
Confusion matrices for the trained k-NN classification model utilizing measured data for different lengths and predictor groups.

Figure 14 illustrates the confusion matrices for the cross-validated classification model, while Figure 15 shows the confusion matrices for the trained k-Nearest Neighbor classifier model.

## 4. Discussion

The examination outcomes were split into two parts: the assessment of the depth and the assessment of the length of the defects.

Before parameter estimation of the depth of natural defects, the classifier underwent training through k-fold cross-validation. Table 8 displays the numerical output of the cost function concerning consecutive sets of predictors. The values of groups D-1b and D-2 were identical, while group D-3 exhibited a comparatively lower value. Figure 11 shows that even for the simulation data cross-validation classification model, for the group of predictors D-1a and D-1b, the correct classification of defect depth poses a significant challenge. The D-2 group demonstrates a high level of accuracy in predicting the majority of cases while consistently encountering an estimation challenge at every level of depth. Group D-3 is facing a minor issue concerning estimating the deepest and cut-through defects.

Following parameter optimization of the simulation-based prediction model, it was subsequently employed to predict actual defects in the measured sample. According to Table 8, the D-2 group exhibited the most accurate prediction of the measurement data, with all defect depths correctly identified. Both Group D-1a and D-3 exhibited similar levels of accuracy in accurately categorizing defects. The confusion matrices for the measurement data of each group are presented in Figure 12. As previously indicated, Group D-2 accurately categorized all levels of imperfections. Despite exhibiting identical loss function values, Groups D-1a and D-3 demonstrate distinct behaviors. Regarding the D-1a group, the error is dispersed among the middle-depth values.

On the other hand, in the D-3 group, the cut-through (100%) defect is inaccurately identified, with a rate of 100%. According to Table 9, the issue with group D-3 pertains to the approximation of measurement data to the theoretical curve outlined in Equation (6). This problem is particularly noticeable for the most extensive defect sizes. Although there is a high level of recognition, Table 9 indicates that the regression $R^{2}$ coefficient of the shallowest defect of 10% is low. This is attributed to the significant waveform distortions caused by measurement noise.

Subsequently, an estimation was made regarding the extent of the defect’s length. Analogously to the aforementioned, Table 10 displays the loss function values for the cross-validation classification model. All predictor groups effectively classified defects using k-fold cross-validation simulation data, as illustrated in Figure 14a. For groups L-1 and L-2, the predicted length’s ultimate value was ascertained by identifying the most frequently occurring value within the set of lengths for subsequent frequencies. Specifically, the length for each component frequency was estimated for a given defect, and the value was selected from among them. The loss function values related to individual frequencies are presented in Table 10 within parentheses and are also visually depicted in Figure 14b (pertaining to the L-1 group) and Figure 14c (pertaining to the L-2 group). The incorporation of the defect depth predictor, which is acquired through the preceding depth estimation stage, is observed to result in a noteworthy improvement in the accurate recognition and classification of length.

Regarding the estimation of length for actual measured data, it was observed that the predictor groups L-1 and L-2 exhibited the minimum value of the loss function, which were 0.33 and 0.25, respectively, as presented in Table 10. The confusion matrices for each case of the predictor group are depicted in Figure 15. Incorporating the depth parameter enhances the accurate identification of the length, as evidenced by the data presented. Even though the loss function in the cross-validation classification model yielded a zero value, groups L-3 and L-4 exhibited comparatively inferior classification outcomes when applied to actual data (Figure 15).

Notably, the penetration depth of eddy currents is comparatively shallow at higher frequencies. Shallow- and deep-seated defects exhibit minimal interaction with eddy currents, resulting in a signal level measured by the sensor that is comparable to the noise level. Consequently, estimating the length of these frequencies is challenging, and in some cases, it may not be feasible. This phenomenon requests consideration in future work.

## 5. Conclusions

This study aimed to apply the k-Nearest Neighbor technique in conjunction with Pulsed Multifrequency Excitation and Spectrogram Eddy Current Testing (PMFES-ECT) to efficiently estimate defect parameters in materials. A 3D simulation model of the measuring sensor was generated to classify defects, and simulated defects with varying parameters, specifically depth and length, were produced. The simulation data was used to train a classification model capable of identifying defects in the tested specimen. The prediction models can be trained using the numerical approach without the need for experimental data.

The findings from the measurements can be summarized as follows. Firstly, favorable outcomes were obtained when employing parameters from groups D-2 and D-3 for depth estimation. However, difficulties were encountered in accurately estimating the defect depth of 100% (cut-through) within group D-3, primarily due to challenges in approximating the measured data points to the predefined function, as indicated by the low $R^{2}$ coefficient of determination. Secondly, the length estimation produced the most effective results for groups L-1 and L-2. Moreover, incorporating prior depth estimation data enhanced the accuracy of defect length recognition.

It should be noted that the sample size used in this investigation was limited. Therefore, employing a broader range of defect types in the sample is recommended for future studies. Additionally, conducting computational simulations encompassing diverse defect profiles would be essential to expand the scope of this detection approach.

## Figures and Tables

**Table 1:** Properties of the INCONEL 600 sample.

Width	Length	Height	Electrical Conductivity	Relative Permeability	Magnetic Properties
165 mm	165 mm	1.25 mm	0.971 MS/m	1.01	Nonmagnetic

**Table 2:** Parameters of the defects manufactured in the INCONEL sample.

Defect Length	Defect Width	Defect Depths
5 mm 7 mm	0.25 mm	1.25 mm (100%, cut-through), 1.00 mm (80%), 0.75 mm (60%), 0.50 mm (40%), 0.25 mm (20%), 0.125 mm (10%)

**Table 3:** Transducer parameters.

Parameter	Value
Excitation coils winding turns	25 turns, Φ 0.14 mm
Measurement coil winding turns	100 turns, Φ 0.02 mm
Core relative permeability	$\mu_{R}=1000$
Maximum working flux density	200 mT

**Table 4:** The groups of predictors and the corresponding predictor variables utilized in each group were used to estimate the depth of a defect.

Predictors Group	Used Predictors
D-1a	$f_{MAX}$, $S_{MAX}$
D-1b	$f_{MAX}$, $S_{MAX}$
D-2	$f_{MAX}$, $\log\left(S_{MAX}\right)$
D-3	α, γ

**Table 5:** The groups of predictors and the corresponding predictor variables utilized in each group were used to estimate the length of a defect.

Predictors Group	Used Predictors
L-1	f, $L_{f}$
L-2	f, $L_{f}$, D
L-3	L
L-4	L, D

**Table 6:** Parameters of the sample plate used in FEM analysis.

Width	Length	Thickness	Electrical Conductivity	Relative Permeability	Magnetic Properties
25 mm	80 mm	1.25 mm	1 MS/m	1.00	Nonmagnetic

**Table 7:** Parameters of defects in the simulated sample.

Defect Length	Defect Width	Defect Depths
1 mm 2 mm 3 mm 4 mm 5 mm 6 mm 7 mm	0.25 mm	1.25 mm (100%, cut-through), 1.00 mm (80%), 0.75 mm (60%), 0.50 mm (40%), 0.25 mm (20%), 0.125 mm (10%)

**Table 8:** Loss function values for the cross-validation classification model (C-V CM) and the prediction classifier losses of defect depth which are categorized by different groups of predictors.

	Predictors Group			
	D-1a	D-1b	D-2	D-3
C-V CM Loss	0.26	0.12	0.12	0.07
Classifier Loss	0.17	0.33	0.00	0.17

**Table 9:** The regression coefficient $R^{2}$ for the fitting of the curve to both simulation (FEM) and measurement (MEAS) data for various depths and groups of predictors.

Defect’s Depth	Predictors Group			
	D-1b & D-2		D-3	
	FEM	MEAS	FEM	MEAS
1.0	0.99	0.99	0.97	0.76
0.8	0.99	0.99	0.96	0.73
0.6	0.99	0.99	0.94	0.80
0.4	0.99	0.99	0.94	0.87
0.2	0.99	0.99	0.94	0.87
0.1	0.99	0.97	0.93	0.72

**Table 10:** Loss function values for the cross-validation classification model (C-V CM) and the prediction classifier losses of defect length which are categorized by different groups of predictors. For the L-1 and L-2 groups, the values of the loss function pertaining to each frequency (treated as a distinct record) are shown in brackets.

	Predictors Group			
	L-1	L-2	L-3	L-4
C-V RM Loss	0.00 (0.10)	0.00 (0.05)	0.00	0.00
Classifier Loss	0.33	0.25	0.42	0.42

## Data Availability

Not applicable.
